# Local Reasoning About the Presence of Bugs: Incorrectness Separation Logic

**DOI:** 10.1007/978-3-030-53291-8_14

**Published:** 2020-06-16

**Authors:** Azalea Raad, Josh Berdine, Hoang-Hai Dang, Derek Dreyer, Peter O’Hearn, Jules Villard

**Affiliations:** 8 Microsoft Research Lab, Redmond, WA USA; 9 University of Southern California, Los Angeles, CA USA; 10 Max Planck Institute for Software Systems (MPI-SWS), Kaiserslautern and Saarbrücken, Germany; 11 Facebook, London, UK; 12 University College London, London, UK

**Keywords:** Program logics, Separation logic, Bug catching

## Abstract

There has been a large body of work on local reasoning for proving the *absence* of bugs, but none for proving their *presence*. We present a new formal framework for local reasoning about the presence of bugs, building on two complementary foundations: 1) separation logic and 2) incorrectness logic. We explore the theory of this new *incorrectness separation logic* (ISL), and use it to derive a begin-anywhere, intra-procedural symbolic execution analysis that has no false positives *by construction*. In so doing, we take a step towards transferring modular, scalable techniques from the world of program verification to bug catching.

## Introduction

There has been significant research on sound, local reasoning about the state for proving the absence of bugs (e.g., [2, 13, 26, 29, 30, 41]). Locality leads to techniques that are compositional *both* in code (concentrating on a program component) and in the resources accessed (spatial locality), without tracking the entire global state or the global program within which a component sits. Compositionality enables reasoning to scale to large teams and codebases: reasoning can be done even when a global program is not present (e.g., a library, or during program construction), without having to write the analogue of a test or verification harness, and the results of reasoning about components can be composed efficiently [11].

Meanwhile, many of the practical applications of symbolic reasoning have aimed at proving the *presence* of bugs (i.e., bug catching), rather than proving their absence (i.e., correctness). Logical bug catching methods include symbolic model checking [7, 12] and symbolic execution for testing [9]. These methods are usually formulated as global analyses; but, the rationale of local reasoning holds just as well for bug catching as it does for correctness: it has the potential to benefit scalability, reasoning about incomplete code, and continuous incremental reasoning about a changing codebase within a continuous integration (CI) system [34]. Moreover, local evidence of a bug without usually-irrelevant contextual information can be more convincing and easier to understand and correct.

There do exist symbolic bug catchers that, at least partly, address scalability and continuous reasoning. Tools such as Coverity [5, 32] and Infer [18] hunt for bugs in large codebases with tens of millions of LOC, and they can even run incrementally (within minutes for small code changes), which is compatible with deployment in CI to detect regressions. However, although such tools intuitively share ideas with correctness-based compositional analyses [16], the existing foundations of correctness-based analyses do not adequately explain what these bug-catchers do, why they work, or the extent to which they work in practice.

A notable such example is the relation between *separation logic* (SL) and Infer. SL provides novel techniques for local reasoning [28], with concise specifications that focus only on the memory accessed [36]. Using SL, symbolic execution need not begin from a “main” program, but rather can “begin anywhere” in a codebase, with constraints on the environment synthesized along the way. When analyzing a component, SL ’s frame rule is used in concert with abductive inference to isolate a description of the memory utilized by the component [11]. Infer was closely inspired by SL, and demonstrates the power of SL ’s local reasoning: the ability to begin anywhere supports incremental analysis in CI, and compositionality leads to highly scalable methods. These features have led to non-trivial impact: a recent paper quotes over 100,000 Infer-reported bugs fixed in Facebook’s codebases, and thousands of security bugs found by a compositional taint analyzer, Zoncolan [18]. However, Infer reports bugs using *heuristics based on failed proofs*, whereas the SL theory behind Infer is based on *over-approximation* [11]. Thus, a critical aspect of Infer’s successful deployment is not supported by the theory that inspired it. This is unfortunate, especially given that the begin-anywhere and scalable aspects of Infer’s algorithms do not appear to be fundamentally tied to over-approximation.

In this paper, we take a step towards transferring the local reasoning techniques from the world of program verification to that of bug catching. To approach the problem from first principles, we do not try to understand tools such as Coverity and Infer as they are. Instead, we take their existence and reported impact as motivation for revisiting the foundations of SL, this time re-casting it as a formalism for proving the *presence* of bugs rather than their absence.

Our new logic, *incorrectness separation logic* (ISL), marries local reasoning based on SL ’s frame rule with the recently-advanced incorrectness logic [35], a formalism for reasoning about errors based on an *under-approximate* analogue of Hoare triples [43]. We observe that the original SL model, based on partial heaps, is incompatible with local, under-approximate reasoning. The problem is that the original model does not distinguish a pointer known to be dangling from one about which we have no knowledge; this in turn contradicts the frame rule for under-approximate reasoning. However, we recover the frame rule for a refined model with negative heap assertions of the form $x \mathrel {\not \mapsto }{}$, read “invalidated *x*”, stating that the location at *x* has been deallocated (and not re-allocated). Negative heaps were present informally in the original Infer, unsupported by theory but added for reporting use-after-free bugs (i.e., not for proving correctness). Interestingly, this semantic feature is needed in ISL for logical (and not merely pragmatic) reasons, in that it yields a *sound* logic for proving the presence of bugs: when ISL identifies a bug, then there is indeed a bug (no false positives), given the assumptions of the underlying ISL model. (That is, as usual, soundness is a relationship between assumptions and conclusions, and whether those assumptions match reality (i.e., running code) is a separate concern, outside the purview of logic.)

As well as being superior for bug reporting, our new model has a pleasant fundamental property in that it meshes better with intuitions originally expressed of SL. Specifically, our model admits a *footprint theorem*, stating that the meaning of a command is solely determined by its transitions on input-output heaplets of minimal size (including only the locations accessed), a theorem that was not true in full generality for the original SL model. Interestingly, ISL supports local reasoning for technically simpler reasons than the original SL (see Sect. 4.2).

We validate part of the ISL promise using an illustrative program analysis, Pulse, and use it to detect *memory safety bugs*, namely null-pointer-dereference and use-after-free bugs. Pulse is written inside Infer [18] and deployed at Facebook where it is used to report issues to C++ developers. Pulse is currently under active development. In this paper, we explore the *intra-procedural* analysis, i.e., how it provides purely local reasoning about one procedure at a time without using results from other procedures; we defer formalising its *inter-procedural* (between procedures) analysis to future work. While leaving out the inter-procedural capabilities of Pulse only partly validates the promise of the ISL theory, it already demonstrates how ISL can scale to large codebases, and run incrementally in a way compatible with CI. Pulse thus has the capability to begin anywhere, and it achieves scalability while embracing under- rather than over-approximation.

**Outline.** In Sect. 2 we present an intuitive account of ISL. In Sect. 3 we present the ISL proof system. In Sect. 4 we present the semantic model of ISL. In Sect. 5 we present our ISL-based Pulse analysis. In Sect. 6 we discuss related work and conclude. The full proofs of all stated theorems are given in the technical appendix [38].

## Proof of a Bug

**Fig. 1.**
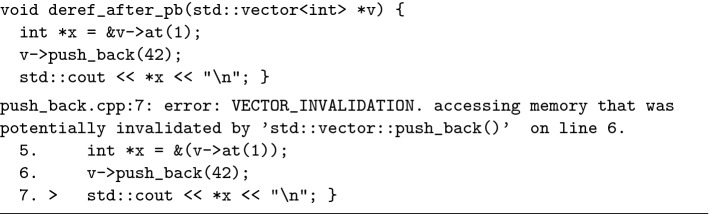
The C++ use-after-lifetime bug (above); the Pulse error message (below).

We proceed with an intuitive description of ISL for detecting memory safety bugs. To do this, in Fig. 1 we present an example of C++ use-after-lifetime bug, abstracted from real occurrences we have observed at Facebook, where use-after-lifetime bugs were one of the leading developer requests for C++ analysis. Given a vector v, a call to push_back(v) in the std::vector library may cause the internal array backing v to be (deallocated and subsequently) reallocated when v needs to grow to accommodate new elements. If the internal array is reallocated during the v->push_back(42) call, a use-after-lifetime bug occurs on the next line as x points into the previous array. Note how the Pulse error message (at the bottom of Fig. 1) refers to memory that has been invalidated. As we describe shortly, this information is tracked in Pulse with an invalidated heap assertion.

**Fig. 2.**
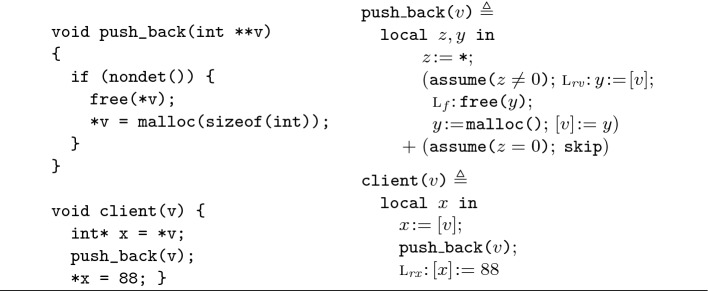
The push_back example in C (left); and in the ISL language (right).

For the theory in this paper, we do not want to descend into the details of C++, vectors, and so forth. Thus, for illustrative purposes, in Fig. 2 we present an adaptation of such use-after-lifetime bugs in C rather than C++, alongside its representation in the ISL language used in this paper. In this adaptation, the array at *v* is of size 1, and is reallocated in push_back non-deterministically to model its dynamic reallocation when growing. We next demonstrate how we can use ISL to detect the use-after-lifetime bug in the client procedure in Fig. 2.

**ISL Triples.** The ISL theory uses *under-approximate triples* [35] of the form , interpreted as: the  assertion describes a *subset* of the states that can be reached from the  assertion by executing $\mathbb {C}$, where $\epsilon$ denotes an *exit condition* indicating either normal or exceptional (erroneous) termination. The under-approximate triples can be equivalently interpreted as: every state in  can be obtained by executing $\mathbb {C}$ on a starting state in . By contrast, given a Hoare triple , the postcondition  describes a *superset* of states that are reachable from the precondition , and may include states unreachable from . Hoare logic is about over-approximation, allowing false positives but not negatives, whereas ISL is about under-approximation, allowing false negatives but not positives.

**Bug Specification of** ${\texttt {client(}}v{\texttt {)}}$**.** Using ISL, we can specify the use-after-lifetime bug in ${\texttt {client(}}v{\texttt {)}}$ as follows:We make several remarks to illustrate the crucial features of ISL:

- As in standard SL, $*$ denotes the separating conjunction, read “and separately”. It implies, e.g., that *v*, $a'$ and *a* are distinct in the result assertion.
- The exit condition $er (\textsc {l}_{ rx })$ denotes an erroneous termination: an error state is reached at line $\textsc {l}_{ rx }$, where *a* is dangling (invalidated).
- The result is under-approximate: any state satisfying the result assertion can be reached from some state satisfying the presumption.
- The specification is local: it focuses only on memory locations in the ${\texttt {client(}}v{\texttt {)}}$ footprint (i.e., those touched by ${\texttt {client(}}v{\texttt {)}}$), and ignores other locations.

Let us next consider how we reason symbolically about this bug. Note that for the ${\texttt {client(}}v{\texttt {)}}$ execution to reach an error at line $\textsc {l}_{ rx }$, the ${\texttt {push\_back(}}v{\texttt {)}}$ call within it must not cause an error. That is, in contrast to PB-Client, we need a specification for ${\texttt {push\_back(}}v{\texttt {)}}$ that describes normal, non-erroneous termination. We specify this normal execution with the  exit condition as follows:PB-Ok describes the case when ${\texttt {push\_back(}}v{\texttt {)}}$ frees the internal array of *v* at *a* (denoted by $a \mathrel {\not \mapsto }{}$ in the result), and subsequently reallocates it at $a'$. Consequently, as *a* is invalidated after the ${\texttt {push\_back(}}v{\texttt {)}}$ call, the instruction following the call in ${\texttt {client(}}v{\texttt {)}}$ dereferences invalidated memory at $\textsc {l}_{rx}$, causing an error.

Note that the result assertion in PB-Ok is strictly under-approximate in that it is smaller (stronger) than the exact “strongest post”. Given the assertion in the presumption, the strongest post must also consider the else clause of the conditional, when nondet() returns zero and ${\texttt {push\_back(}}v{\texttt {)}}$ does nothing. That is, the strongest post is the disjunction of the given result and the presumption. The ability to go below the strongest post soundly is a hallmark of under-approximate reasoning: it allows for compromise in an analyzer, where we might choose, e.g., to limit the number of paths explored for efficiency reasons, or to concretize an assertion partially when symbolic reasoning becomes difficult [35].

**Fig. 3.**
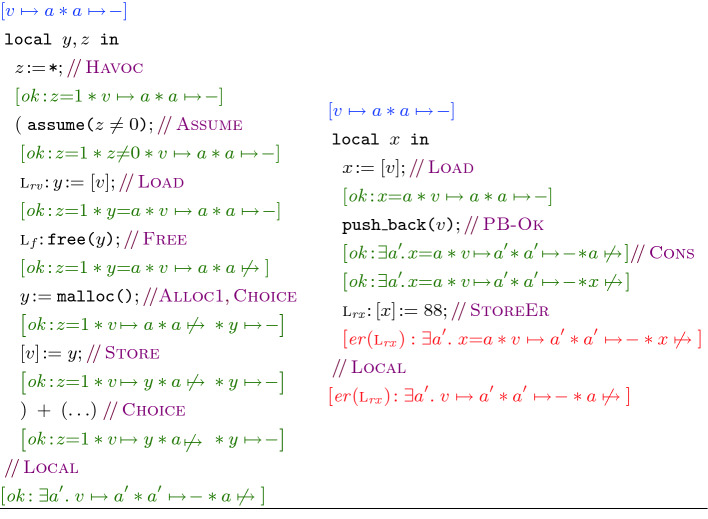
The proof sketches of PB-Ok (left) and PB-Client (right).

We present proof outlines for PB-Ok and PB-Client in Fig. 3, where we annotate each step with a proof rule to connect to the ISL theory in Sect. 3. For legibility, uses of the Frame rule are omitted as it is used in almost every step, and the consequence rule Cons is usually omitted when rewriting a formula to an equivalent one. For the moment, we encourage the reader to attempt to follow, prior to formalization, by mentally executing the program instructions on the assertions and asking: does the assertion at each program point under-approximate the states that can be obtained from the prior state? Note that each step updates assertions in-place, just as concrete execution does on concrete memory. For example, $\textsc {l}_f{:}\,\texttt {free(}y\texttt {)}$ replaces $a \mapsto {\!-}$ with $a \mathrel {\not \mapsto }{}$. In-place reasoning is a capability that the separating conjunction brings to symbolic execution; formally, this in-place aspect is achieved in the logic by applying the frame rule.

## Incorrectness Separation Logic (ISL)

As a first attempt, it is tempting to obtain ISL straightforwardly by composing the standard semantics of SL [41] and the semantics of incorrectness logic [35]. Interestingly, this simplistic approach does not work. To see this, consider the following axiom for freeing memory, adapted from the corresponding SL axiom:Here, $\mathsf {emp}$ describes the empty heap and $\mathtt {loc}(x)$ states that *x* is an addressable location; e.g., *x* cannot be null. Note that this ISL triple is valid in that any state satisfying the result assertion can be obtained from one satisfying the presumption assertion, and thus we do have a true under-approximate triple.

However, in SL one can arbitrarily extend the state using the frame rule:Intuitively, the state described by the *frame* assertion *r* lies outside the footprint of $\mathbb {C}$ and thus remains unchanged when executing $\mathbb {C}$. However, if we do this with the $\texttt {free(}x\texttt {)}$ axiom above, choosing $x \mapsto {-}$ as our frame, we run into a problem:Here, the presumption is inconsistent but the result is not, and thus there is no way to get back to the presumption from the result; i.e., the triple is invalid. In over-approximate reasoning this does not cause a problem since an inconsistent precondition renders an over-approximate triple vacuously valid. By contrast, an inconsistent presumption does not validate under-approximate reasoning.

Our way out of this conundrum is to consider a modified model in which the knowledge that a location was previously freed is a resource-oriented fact, using negative heap assertions. The negative heap assertion $x \mathrel {\not \mapsto }{}$ conveys more knowledge than the $\mathtt {loc}(x)$ assertion. Specifically, $x \mathrel {\not \mapsto }{}$ conveys: 1) the *knowledge* that *x* is an addressable location; 2) the knowledge that *x* has been deallocated; and 3) the *ownership* of location *x*. In other words, $x \mathrel {\not \mapsto }{}$ is analogous to the points-to assertion $x \mapsto {-}$ and is thus manipulated similarly, taking up space in $*$-conjuncts. That is, we cannot consistently $*$-conjoin $x \mathrel {\not \mapsto }{}$ either with $x \mapsto {-}$ or with itself: $x \mapsto {-} * x \mathrel {\not \mapsto }{} \Leftrightarrow \mathsf {false}$ and $x \mathrel {\not \mapsto }{} * x \mathrel {\not \mapsto }{} \Leftrightarrow \mathsf {false}$.

With such negative assertions, we can specify $\texttt {free(}$ ) as the Free axiom in Fig. 5. Note that this allows us to recover the frame rule: when we frame $x \mapsto {-}$ on both sides, we obtain the inconsistent assertion $x \mapsto {-} * x \mathrel {\not \mapsto }{}$ (i.e., $\mathsf {false}$) in the result, which always makes an under-approximate triple vacuously valid.

We demonstrated how we arrived at negative heaps as a theoretical solution to recover the frame rule. However, negative heaps are more than a technical curiosity. In particular, a similar idea was informally present in Infer and has been used formally to reason about JavaScript [21]. Moreover, as we show in Sect. 4, negative heaps give rise to a *footprint theorem* (see Theorem 2).

Negative heap assertions were previously used informally in Infer. They were also independently and formally introduced in a separation logic for JavaScript [21] to state that a field is not present in a JavaScript object, which is a natural property to express when reasoning about JavaScript.

**Fig. 4.**
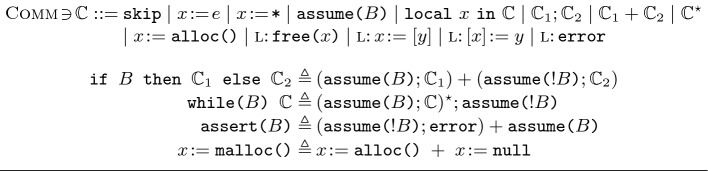
The ISL Language (above); encoding standard constructs in ISL (below).

**Programming Language.** To keep our presentation concise, we employ a simple heap-manipulating language as shown in Fig. 4. We assume an infinite set $\textsc {Val}$ of *values*; a finite set $\textsc {Var}$ of (program) *variables*; a standard interpreted language for *expressions*, $\textsc {Exp}$, containing variables and values; and a standard interpreted language for *Boolean expressions*, $\textsc {BExp}$. We use *v* as a metavariable for values; *x*, *y*, *z* for program variables; *e* for expressions; and *B* for Boolean expressions.

Our language is given by the $\mathbb {C}$ grammar and includes the standard constructs of skip, assignment ($x\!:=\!e$), non-deterministic assignment ($x\!:=\!{\texttt {*}}$, where ${\texttt {*}}$ denotes a non-deterministically picked value), assume statements (${\texttt {assume(}}B{\texttt {)}}$), scoped variable declaration (${\texttt {local}}\;x \;{\texttt {in}}\; \mathbb {C}$), sequential composition ($\mathbb {C}_1; \mathbb {C}_2$), non-deterministic choice ($\mathbb {C}_1 + \mathbb {C}_2$) and loops ($\mathbb {C}^{\star }$), as well as error statements (error) and heap-manipulating instructions. Note that deterministic choice and loops (e.g., if and while statements) can be encoded using their non-deterministic counterparts and assume statements, as shown in Fig. 4.

To better track errors, we annotate instructions that may cause an error with a label $\textsc {l}\!\in \! \textsc {Label}$. When an error is encountered (e.g., in l: error), we report the label of the offending instruction (e.g., l). As such, we only consider *well-formed* programs: those with unique labels across their constituent instructions. For brevity, we drop the instruction labels when they are immaterial to the discussion.

As is standard practice, we use error statements as test oracles to detect violations. In particular, error statements can be used to encode *assert* statements as shown in Fig. 4. Heap-manipulating instructions include allocation, deallocation, lookup and mutation. The $x\!:={\texttt {alloc()}}$ instruction allocates a new (unused) location on the heap and returns it in *x*, and can be used to represent the standard, possibly ${\texttt {null}}$-returning ${\texttt {malloc()}}$ from C as shown in Fig. 4. Dually, $\texttt {free(}x\texttt {)}$ deallocates the location denoted by *x*. Heap lookup $x\!:=[y]$ reads the contents of the location denoted by *y* and returns it in *x*; heap mutation $[x]\!:=y$ overwrites the contents of the location denoted by *x* with *y*.

**Assertions.** The *ISL assertion language* is given by the grammar below, where $\oplus \!\in \! \{=, \ne , <, \le , \ldots \}$. We use *p*, *q*, *r* as metavariables for assertions.As we describe formally in Sect. 4, assertions describe sets of *states*, where each state comprises a (variable) store and a heap. The classical (first-order logic) and Boolean assertions are standard. Other classical connectives can be encoded using existing ones (e.g., $\lnot p \triangleq p \Rightarrow \mathsf {false}$). Aside from the  $x \mathrel {\not \mapsto }{}$, structural assertions are as defined in SL [28], and describe a set of states by constraining the shape of the underlying heap. More concretely, $\mathsf {emp}$ describes states in which the heap is empty; $e \mapsto {e'}$ describes states in which the heap comprises a single location denoted by *e* containing the value denoted by $e'$; and $p * q$ describes states in which the heap can be split into two disjoint sub-heaps, one satisfying *p* and the other *q*. We often write $e \mapsto {-}$ as a shorthand for $\exists v.\; e \mapsto {v}$.

As described above, we extend our structural assertions with the *negative* heap assertion $e \mathrel {\not \mapsto }{}$ (read “*e* is invalidated”). As with its positive counterpart $e \mapsto {e'}$, the negative assertion $e \mathrel {\not \mapsto }{}$ describes states in which the heap comprises a single location at *e*. However, whilst $e \mapsto {e'}$ states that the location at *e* is allocated (and contains the value $e'$), $e \mathrel {\not \mapsto }{}$ states that the location at *e* is *deallocated*.

**Fig. 5.**
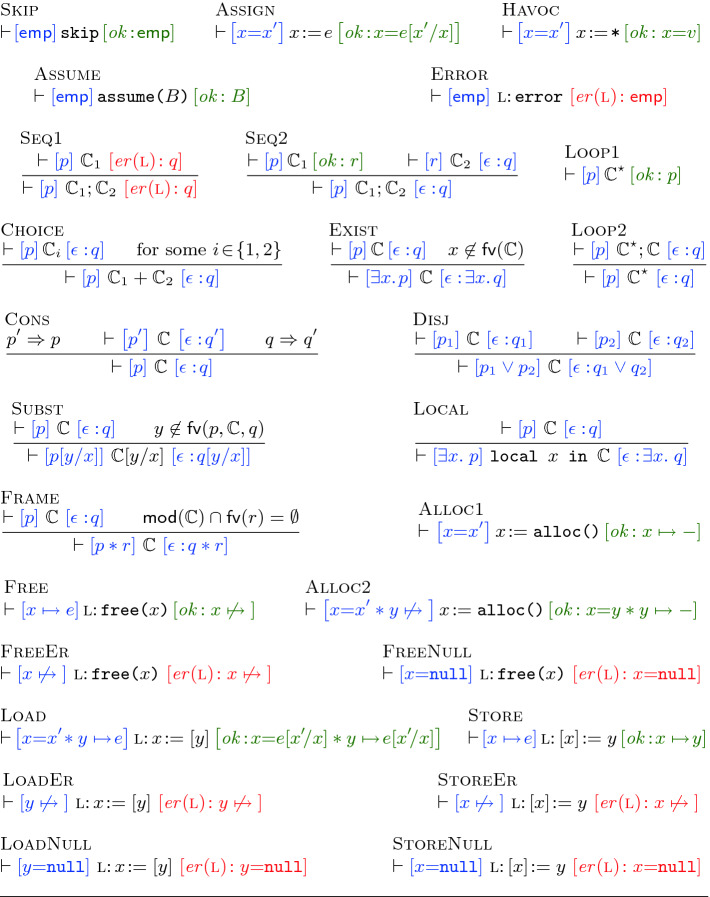
The ISL proof rules where *x* and $x'$ are distinct variables.

**ISL Proof Rules (Syntactic ISL Triples).** We present the ISL proof rules in Fig. 5. As in incorrectness logic [35], the ISL triples are of the form , denoting that *every* state in the *result* assertion *q* is reachable from *some* state in the *presumption* assertion *p* with *exit condition* $\epsilon$. That is, for each state $\sigma _q$ in *q*, there exists $\sigma _p$ in *p* such that executing $\mathbb {C}$ on $\sigma _p$ terminates with $\epsilon$ and yields $\sigma _q$. As such, since $\mathsf {false}$ describes an empty state set,  is vacuously valid for all *p*, $\mathbb {C}$, $\epsilon$. Dually,  is always invalid when $q \not \Rightarrow \mathsf {false}$.

An exit condition, $\epsilon \in \textsc {Exit}$, may be: 1) $ok$, denoting a successful execution; or 2) $er (\textsc {l})$, denoting an erroneous execution with the error encountered at the $\textsc {l}$-labeled instruction. Compared to [35], we further annotate our error conditions to track the offending instructions. Moreover, whilst [35] rules only detect explicit errors caused by error statements, ISL rules additionally allow us to track errors caused by *memory safety violations*, namely “use-after-free” violations, where a previously deallocated location is subsequently accessed in the program, and “null-pointer-dereference” violations. Although it is straightforward to distinguish between explicit and memory safety errors, for brevity we use $er (\textsc {l})$ for both.

Thanks to the separation afforded by ISL assertions, compared to incorrectness triples in [35], ISL triples are *local* in that the states described by their presumptions only contain the resources needed by the program. For instance, as skip requires no resource for successful execution, the presumption of Skip is simply given by $\mathsf {emp}$, which remains unchanged in the result. Similarly, ${\texttt {assume(}}B{\texttt {)}}$ requires no resource and results in a state satisfying *B*. The Assign rule is analogous to its SL counterpart. Similarly, $x\!:={\texttt {*}}$ in Havoc assigns a non-deterministic value to *x*. Although these axioms (and Alloc1, Alloc2) ask for a single equality $x=x'$ in their presumption, one can derive more general triples starting from any presumption *p* by picking a fresh $x'$ and applying the axiom, and the Frame and Cons rules on the equivalent presumption $x=x' * p[x'/x]$.

Note that ${\texttt {skip}}$, assignments and assume statements always terminate successfully (with $ok$). By contrast, $\textsc {l}{:}\,{\texttt {error}}$ always terminates erroneously (with $er (\textsc {l})$) and requires no resource. The ISL rules Seq1, Seq2, Choice, Loop1, Loop2, Cons, Disj and Subst are as in [35]. The Seq1 rule captures short-circuiting when the first statement ($\mathbb {C}_1$) encounters an error and thus the program terminates erroneously. Analogously, Seq2 states that when $\mathbb {C}_1$ executes successfully, the program terminates with $\epsilon$ when the subsequent $\mathbb {C}_2$ statement terminates with $\epsilon$. The Choice rule states that the states in *q* are reachable from *p* when executing $\mathbb {C}_1 + \mathbb {C}_2$ if they are reachable from *p* when executing either branch. Loop1 captures immediate exit from the loop; Loop2 states that *q* is reachable from *p* when executing $\mathbb {C}^{\star }$ if it is reachable after a non-zero number of $\mathbb {C}$ iterations.

The Cons rule allows us to strengthen the result and weaken the presumption: if $q'$ is reachable from $p'$, then the smaller *q* is reachable from the bigger *p*. Note that compared to SL, the direction of implications in the Cons premise are flipped. Using Cons, we can rewrite the premises of Disj as  and . As such, if both $q_1$ and $q_2$ are reachable from $p_1 \vee p_2$, then $q_1 \vee q_2$ is also reachable from $p_1 \vee p_2$, as shown in Disj. The Exist rule is derived from Disj; Subst is standard and allows us to substitute *x* with a fresh variable *y*; Local is equivalent to that in [35] but uses the Barendregt variable convention, renaming variables in formulas instead of in commands to avoid clashes.

As in SL, the crux of ISL reasoning lies in the Frame rule, allowing one to extend the presumption and the result of a triple with disjoint resources in *r*. The $\mathsf {fv}(r)$ function returns the set of free variables in *r*, and $\mathsf {mod}(\mathbb {C})$ returns the set of (program) variables modified by $\mathbb {C}$ (i.e., those on the left-hand of ‘:=’ in assignment, lookup and allocation). These definitions are standard and elided.

Negative assertions allow us to detect memory safety violations when accessing deallocated locations. For instance, FreeEr states that attempting to deallocate *x* causes an error when *x* is already deallocated; *mutatis mutandis* for LoadEr and StoreEr. As shown in Alloc2, we can use negative assertions to allocate a previously-deallocated location: if *y* is deallocated ($y \mathrel {\not \mapsto }{}\!$ holds in the presumption), then it may be reallocated. The FreeNull, LoadNull and StoreNull rules state that accessing *x* causes an error when *x* is null. Finally, Load and Store describe the successful execution of heap lookup and mutation, respectively.

### Remark 1

Note that mutation and deallocation rules in SL are given as  and ; i.e., the value of *x* is existentially quantified in the precondition. We can similarly rewrite the ISL rules as:However, these rules are too weak. For instance, we cannot use StoreWeak to prove . This is because the implications in the premise of the Cons rule are flipped from those in their SL counterpart, and thus to use StoreWeak we must show $x \mapsto {-} \Rightarrow x \mapsto {7}$ which we cannot. Put differently, StoreWeak states that for *some* value *v*, executing $[x]\!:=y$ on a state satisfying $x \mapsto {v}$ yields a state satisfying $x \mapsto {y}$. However, this statement is valid for *all* values of *v*. As such, we strengthen the presumption of Store to $x \mapsto {e}$, allowing for an arbitrary (universally quantified) expression *e* at *x*.

In general, in over-approximate logics (e.g., SL) the aim is to *weaken* the preconditions and *strengthen* the postconditions of specifications as much as possible. This is to ensure that we can optimally apply the Cons rule to adapt the specifications to broader contexts. Conversely, in under-approximate logics (e.g., ISL) we should strengthen the presumptions and weaken the results as much as possible, since the implication directions in the premise of Cons are flipped.

### Remark 2

The backward reasoning rules of SL [28] are generally unsound for ISL, just as the backward reasoning rules of Hoare logic are unsound for incorrectness logic [35]. For instance, the backward axiom for store is . However, taking $p=\mathsf {emp}$ yields an inconsistent precondition, resulting in the triple , which is valid in SL but not ISL.

**Proving.** PB-Ok **and** PB-Client**.** We next return to the proof sketch of PB-Ok in Fig. 3. For brevity, rather than giving full derivations, we follow the classical Hoare logic proof outline, annotating each line of the code with its presumption and result. We further commentate each proof step and write e.g., //Choice to denote an application of Choice. Note that when applying Choice, we *pick* a branch (e.g., the left branch in PB-Ok) to execute. Observe that unlike in SL where one needs to reason about *all* branches, in ISL it suffices to pick and reason about a *single* branch, and the remaining branches are ignored.

**Fig. 6.**
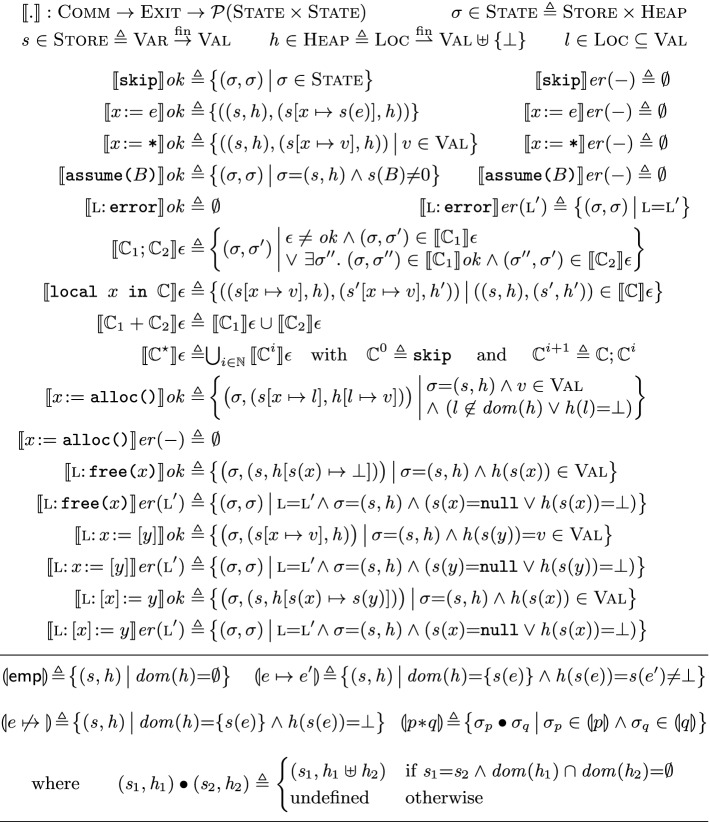
The ISL denotational semantics (top); the ISL assertion semantics (bottom).

As in Hoare logic proof outlines, we assume that Seq2 is applied at every step; i.e., later instructions are executed only if the earlier ones execute successfully. In most steps, we apply Frame to frame off the unused resource *r*, carry out the instruction effect, and subsequently frame on *r*. For instance, when verifying $z\!:={\texttt {*}}$ in the proof sketch of PB-Ok, we apply Havoc to pick a non-zero value for *z* (in this case 1) after the assignment. As such, since the presumption of Havoc is $\mathsf {emp}$, we use Frame to frame off the resource $v \mapsto {a} * a \mapsto {\!-}$ in the presumption, apply Havoc to obtain $z = 1$, and subsequently frame on $v \mapsto {a} * a \mapsto {\!-}$, yielding $z = 1 * v \mapsto {a} * a \mapsto {\!-}$. For brevity, we keep the applications of Frame and Seq2 implicit and omit them in our annotations. The proof of PB-Client in Fig. 3 is then straightforward and applies the PB-Ok specification when calling ${\texttt {push\_back(}}v{\texttt {)}}$. We refer the reader to the technical appendix [38] where we apply ISL to a further example to detect a null-pointer-dereference bug in OpenSSL.

## The ISL Model

**Denotational Semantics.** We present the ISL semantics in Fig. 6. The semantics of a statement $\mathbb {C}\in \text {Comm}$ under an exit condition $\epsilon \in \textsc {Exit}$, written $\llbracket \mathbb {C}\rrbracket \epsilon$, is described as a relation on *program states*. A program state, $\sigma \in \textsc {State}$, is a pair of the form $( s , h )$, comprising a (variable) *store* $s \in \textsc {Store}$ and a *heap* $h \in \textsc {Heap}$.

A store is a function from variables to values. Given a store $$ s $$, expression *e* and Boolean expression *B*, we write $$ s (e)$$ and $$ s (B)$$ for the values to which *e* and *B* evaluate under $$ s $$, respectively. These definitions are standard and omitted.

A heap is a partial function from *locations*, $$\textsc {Loc}$$, to $$\textsc {Val}\uplus \{\bot \}$$. We model heaps as partial functions as they may grow gradually by allocating additional locations. We use the designated value $$\bot \not \in \textsc {Val}$$ to track those locations that have been deallocated. That is, given $$l \in \textsc {Loc}$$, if $$ h (l) \in \textsc {Val}$$ then *l* is allocated in *h* and holds value $$ h (l)$$; and if $$ h (l) = \bot $$ then *l* has been deallocated. As we demonstrate shortly, we use $$\bot $$ to model invalidated assertions such as $$x \mathrel {\not \mapsto }{}$$.

The semantics in Fig. 6 closely corresponds to ISL rules in Fig. 5. For instance, $\llbracket x\!:=[y]\rrbracket ok$ underpins Load, while $\llbracket x\!:=[y]\rrbracket er (-)$ underpins LoadEr and LoadNull; e.g., if the location at *y* is deallocated ($h ( s (y)) \!=\! \bot$), then executing $x\!:=[y]$ terminates erroneously as captured by $\llbracket x\!:=[y]\rrbracket er (-)$. The semantics of mutation, allocation and deallocation are defined analogously. As shown, skip, assignment and ${\texttt {assume(}}B{\texttt {)}}$ never terminate erroneously (e.g., $\llbracket {\texttt {skip}} \rrbracket er (-) \!=\! \emptyset$), and the semantics of their successful execution is standard. The two disjuncts in $\llbracket \mathbb {C}_1; \mathbb {C}_2\rrbracket \epsilon$ capture Seq1 and Seq2, respectively. The semantics of $\mathbb {C}_1 + \mathbb {C}_2$ is defined as the union of those of its two branches. The semantics of $\mathbb {C}^{\star }$ is defined as the union of the semantics of zero or more $\mathbb {C}$ iterations.

**Heap Monotonicity.** Note that for all $\mathbb {C}$, $\epsilon$ and $(\sigma _p, \sigma _q) \in \llbracket \mathbb {C}\rrbracket \epsilon$, the (domain of the) underlying heap in $\sigma _p$ *monotonically grows* from $\sigma _p$ to $\sigma _q$ and *never shrinks*. In particular, whilst the heap domain grows via allocation, all other base cases (including deallocation) leave the domain of the heap (i.e., the heap size) unchanged – deallocation merely updates the value of the given location in the heap to $\bot$ and thus does not alter the heap domain. This is in contrast to the original SL model [28], where deallocation *removes* the given location from the heap, and thus the underlying heap may grow or shrink. As we discuss shortly, this monotonicity is the key reason why our model supports a footprint theorem.

**ISL Assertion Semantics.** The *semantics of ISL assertions* is given at the bottom of Fig. 6 via the function , interpreting each assertion as a set of states. The semantics of classical and Boolean assertions are standard and omitted. As described in Sect. 3, $$\mathsf {emp}$$ describes states in which the heap is empty; and $$e \mapsto {e'}$$ describes states of the form $$( s , h )$$ in which $$ h $$ contains a single location at $$ s (e)$$ with value $$ s (e')$$. Analogously, $$e \mathrel {\not \mapsto }{}$$ describes states of the form $$( s , h )$$ in which $$ h $$ contains a single deallocated location at $$ s (e)$$. Finally, the interpretation of $$p*q$$ contains a state $$\sigma $$ iff it can be split into two parts, $$\sigma = \sigma _p \bullet \sigma _q$$, such that $$\sigma _p$$ and $$\sigma _q$$ are included in the interpretations of *p* and *q*, respectively. The function $$\bullet : \textsc {State}\times \textsc {State}\rightharpoonup \textsc {State}$$ given at the bottom of Fig. 6 denotes *state composition*, and is defined when the constituent stores agree and the heaps are disjoint. For brevity, we often write $$\sigma \in p$$ for .

**Semantic Incorrectness Triples.** We next present the formal interpretation of ISL triples. Recall from Sect. 3 that an ISL triple  states that every state in *q* is reachable from some state in *p* under $\epsilon$. Put formally:Finally, in the following theorem we show that the ISL proof rules are *sound*: if a triple  is derivable using the rules in Fig. 5, then  holds.

### Theorem 1

**(Soundness).** For all , if , then .

### The Footprint Theorem

The frame rule of SL enables *local* reasoning about a command $\mathbb {C}$ by concentrating only on the parts of the memory that are accessed by $\mathbb {C}$, i.e., the $\mathbb {C}$ *footprint*:

‘To understand how a program works, it should be possible for reasoning and specification to be confined to the cells that the program actually accesses. The value of any other cell will automatically remain unchanged.’ [36]

Local reasoning is then enabled by semantic observations about the local effect of heap accesses. In what follows we describe some of the semantic structure underpinning under-approximate local reasoning, including how it differs from the classic over-approximate theory. Our main result is a footprint theorem, stating that the meaning of a command $\mathbb {C}$ is determined by its action on the “small” part of the memory accessed by $\mathbb {C}$ (i.e., the $\mathbb {C}$ footprint). The overall meaning of $\mathbb {C}$ can then be obtained by “fleshing out” its footprint.

To see this, consider the following example: 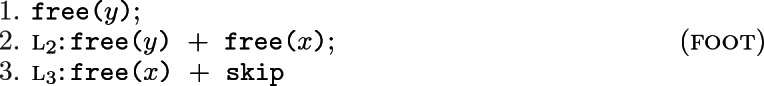
For simplicity, let us ignore variable stores for the moment and consider the executions of foot from an initial heap $h \triangleq [l_x \mapsto 1 , l_y \mapsto 2, l_z \mapsto 3]$, containing locations $l_x$, $l_y$ and $l_z$, corresponding to variables *x*, *y* and *z*, respectively. Note that starting from $h$, foot gives rise to four executions depending on the $+$ branches taken at lines 2 and 3. Let us consider the successful execution from $h$ that first frees *y*, then frees *x* (the right branch of $+$ on line 2), and finally executes skip (the right branch of $+$ on line 3). The footprint of this execution from $h$ is then given by $( ok : [l_x \mapsto 1 , l_y \mapsto 2], [l_x \mapsto \bot , l_y\mapsto \bot ])$, denoting an $ok$ execution from the initial sub-heap $[l_x \mapsto 1 , l_y \mapsto 2]$, yielding the final sub-heap $[l_x \mapsto \bot , l_y \mapsto \bot ]$ upon termination. That is, the initial and final sub-heaps in the footprint do not include the untouched location $l_z$ as it remains unchanged, and the overall effect of foot is obtained from its footprint by adding $l_z \mapsto 3$ to both the initial and final sub-heaps; i.e., by “fleshing out” the footprint.

Next, consider the execution in which the left branch of $+$ on line 2 is taken, resulting in a use-after free error. The footprint of this second execution from $h$ is given by $( er (\textsc {l}_{2}): [l_y \mapsto 2], [l_y \mapsto \bot ])$, denoting an error at $\textsc {l}_{2}$. Note that as this execution terminates erroneously at $\textsc {l}_{2}$, unlike in the first execution, location $l_x$ remains untouched by foot and is thus not included in the footprint.

**Fig. 7.**
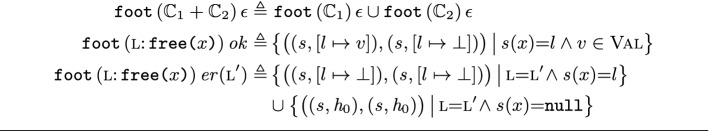
The $$\mathtt {foot}\left( .\right) $$ function (excerpt), where $$ h _0$$ denotes an empty heap ($$ dom ( h _0) \,{=}\, \emptyset $$).

Put formally, let $$\mathtt {foot}\left( .\right) : \text {Comm}\rightarrow \textsc {Exit}\rightarrow \mathcal {P}(\textsc {State}\times \textsc {State})$$ denote a *footprint function* such that $$\mathtt {foot}\left( \mathbb {C}\right) \epsilon $$ describes the *minimal* state needed for *some* $$\mathbb {C}$$ execution under $$\epsilon $$: if $$(( s , h ), ( s ', h ')) \!\in \! \mathtt {foot}\left( \mathbb {C}\right) \epsilon $$, then $$ h $$ contains only the locations accessed by some $$\mathbb {C}$$ execution, yielding $$ h '$$ on termination. In Fig. 7 we present an excerpt of $$\mathtt {foot}\left( .\right) $$, with its full definition given in [38]. Our footprint theorem (Theorem 2) then states that any pair $$(\sigma _p, \sigma _q)$$ resulting from executing $$\mathbb {C}$$ (i.e., $$(\sigma _p, \sigma _q) \in \llbracket \mathbb {C}\rrbracket \epsilon $$) can be obtained by fleshing out a pair $$(\sigma '_p, \sigma '_q)$$ in the $$\mathbb {C}$$ footprint (i.e., $$(\sigma '_p, \sigma '_q) \in \mathtt {foot}\left( \mathbb {C}\right) \epsilon $$): $$(\sigma _p, \sigma _q) = (\sigma '_p \bullet \sigma _r, \sigma '_q \bullet \sigma _r)$$ for some $$\sigma _r$$.

#### Theorem 2

**(Footprints).** For all $\mathbb {C}$ and $\epsilon$: $\llbracket \mathbb {C}\rrbracket \epsilon = \mathtt {frame}\left( \mathtt {foot}\left( \mathbb {C}\right) \epsilon \right)$, where .

We note that our footprint theorem is a positive by-product of the ISL *model* and *not* the ISL logic. That is, the footprint theorem is an added bonus of the heap monotonicity in the ISL model, brought about by negative heap resources, and is orthogonal to the notion of under-approximation. As such, the footprint theorem would be analogously valid in the original SL model, were we to alter its model to achieve heap monotonicity through negative heaps. That said, there are important differences with the classic SL theory, which we discuss next.

### Differences with the Classic (Over-Approximate) Theory

Existing work [14, 40] presents footprint theorems for classical SL based on the notion of *safe states*; i.e., those that do not lead to erroneous executions. This is understandable as the informal reasoning which led to the frame rule for SL was based on safety [36, 45]. According to the *fault-avoiding interpretation* of an SL triple , deemed invalid when a state in *p* leads to an error, if $\mathbb {C}$ accesses a location outside *p*, then this leads to a safety violation. As such, any location not guaranteed to exist in *p* must remain unchanged, thereby yielding the frame rule. The existing footprint theorems were for safe states only.

By contrast, our theorem considers footprints involving both unsafe and safe states. For instance, given the foot program and an initial state (e.g., $h$ in Sect. 4.1), we distinguished a footprint leading to an erroneous execution (e.g., $( er (\textsc {l}_{2}): [l_y \mapsto 2], [l_y \mapsto \bot ])$) from one leading to a safe execution (e.g., $( ok : [l_x \mapsto 1 , l_y \mapsto 2], [l_x \mapsto \bot , l_y\mapsto \bot ])$). This distinction is important, as otherwise we could not distinguish further bugs that follow a safe execution. To see this, consider a second error in foot, namely the possible use-after-free of *x* on line 3, following a successful execution of lines 1 and 2.

For reasoning about incorrectness, it is essential that we consider unsafe states when accounting for why things work; this is a technical difference with the classic footprint results. But it also points to a deeper conceptual difference between the correctness and incorrectness theories. Above, we explained how safety, and its violation, played a crucial role in justifying the frame rule of over-approximate SL. However, as we describe below, ISL and its frame rule do not rely on safety.

As shown in [35], an under-approximate triple can be equivalently defined as: , where $\mathsf {post}(\mathbb {C}, p)$ describes the states obtained by executing $\mathbb {C}$ on *p*. While this under-approximate definition equivalently justifies the frame rule, the analogous over-approximate (Hoare) triple obtained by flipping $\supseteq$ (i.e., ) invalidates the frame rule:The premise of this derivation is valid according to the standard interpretation of over-approximate triples, but its conclusion (obtained by framing on $x \mapsto {17}$) certainly is not, as it states that the value of *x* remains unchanged after mutation.

The frame rule is then recovered by strengthening the  interpretation, *either* by requiring that executing $\mathbb {C}$ on *p* not fault (fault avoidance), *or* by “baking in” frame preservation: $\forall r.\; \mathsf {post}(\mathbb {C}, p*r) \subseteq q * r$. Both solutions then invalidate the premise of the above derivation. We found it remarkable that our ISL theory is consistent with the technically simpler interpretation of triples – namely as $\mathsf {post}(\mathbb {C}, p) \supseteq q$, the dual of Hoare’s interpretation – and that it supports a simple footprint theorem at once, again in contrast to the over-approximate theory.

## Begin-Anywhere, Intra-procedural Symbolic Execution

ISL lends itself naturally to the definition of forward symbolic execution analyses. We demonstrate that using the ISL rules, it is straightforward to derive a *begin-anywhere*, *intra-procedural* analysis that allows us to infer valid ISL triples *automatically* for a given piece of code, with the goal of finding only true bugs reachable from an initial state. This is implemented in the intra-procedural-only mode of the Pulse analysis inside Infer [18] (accessible by passing ––pulse ––pulse-intraprocedural-only to infer). The analysis follows principles from bi-abduction [11], but takes its most successful application – bug catching [18] – as the sole objective. This allows us to make a number of adjustments and to obtain an analysis that is a much closer fit to the ISL theory of under-approximation than the original bi-abduction analysis was to the SL theory of over-approximation.

The original bi-abduction analysis in Abductor [11] and Infer [18] aimed at discovering fault-avoiding specifications for procedures. Ideally, one would find specifications for *all* procedures in the codebase, all the way to an entry-point (e.g., the main() function), thus proving the program safe. In practice, however, virtually all sizable codebases have bugs, and known abstract domains are imprecise when proving memory safety for large codebases. As such, specifications were found for only 40–70% of the procedures in the experiments of [11]. Nonetheless, proof failures, a by-product of proof search, became practically more valuable than proofs, as they can indicate errors. Complex heuristics came into play to classify proof failures and to report to the programmer those more likely to be errors. These heuristics have not been given a formal footing, contributing to the gap between the theory of proofs and the practice of bug catching.

Pulse approaches bug reporting more directly: by looking for them. It infers under-approximate specifications, while recording invalidated addresses. If such an address is later accessed, a bug is reported soundly, in line with the theory.

**Fig. 8.**
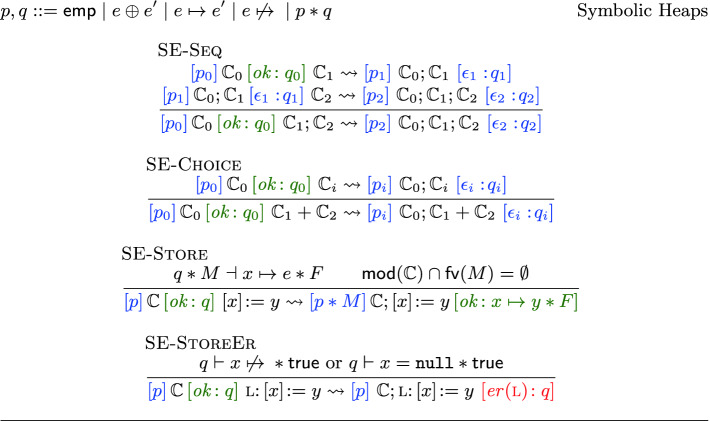
Symbolic heaps (above) and selected symbolic execution rules (below).

**Symbolic Execution.** In Fig. 8 we present our symbolic execution as big-step, syntax-directed inference rules of the form , which can be read as: “having already executed $\mathbb {C}_0$ yielding (discovering) the presumption $p_0$ and the result $q_0$, then executing $\mathbb {C}$ yields the presumption *p* and result *q*”. As is standard in SL-based tools [4, 11], our abstract states consist of $*$-conjoined predicates, with the notable addition of the invalidated assertion and omission of inductive predicates. The latter are not needed because we never perform the over-approximation steps that would introduce them.

SE-Seq describes how the symbolic execution goes forward step by step. SE-Choice describes how the analysis computes one specification per path taken in the program. To ensure termination, loops are unrolled up to a fixed bound $N_\text {loops}$, borrowing from symbolic bounded model checking [12]. These two ideas avoid the arduous task of inventing join and widen operators [15]. For added efficiency, in practice we also limit the maximum number of paths leading to the same program point to a fixed bound $N_\text {disjuncts}$. The $N_\text {loops}$ and $N_\text {disjuncts}$bounds give us easy “knobs” to tune the precision of the analysis. Note that pruning paths by limiting disjuncts is also sound for under-approximate reasoning [35].

To analyze a program $\mathbb {C}$, we start from $\mathbb {C}_0 ={\texttt {skip}}$ and produce . As  holds and symbolic execution rules preserve validity, we then obtain valid triples for $\mathbb {C}$ by Theorem 3.

### Theorem 3

**(Soundness of Symbolic Execution).** If  and , then .

Symbolic execution of individual commands follows the derived SymbExec rule below, with the side-condition that $\mathsf {mod}(\mathbb {C}_0) \cap \mathsf {fv}(M) = \mathsf {mod}(\mathbb {C}) \cap \mathsf {fv}(F) = \emptyset$:If executing $\mathbb {C}_0$ yields the presumption $p_0$ and the current state $q_0$, then SymbExec allows us to execute the next command $\mathbb {C}$ with specification . This may 1) materialize a state *M* that is *missing* from $q_0$ (and is needed to execute $\mathbb {C}$); and 2) carry over an unchanged *frame* *F*. The unknowns *M* and *F* in the *bi-abduction question* $p * F \vdash q_0 * M$ have analogous counterparts in over-approximate bi-abduction; but, as in the Cons rule, their roles have flipped: the *frame* *F* is *abduced*, while the missing *M* is *framed* (or *anti-abduced*).

**Bi-abduction and ISL.** Bi-abduction is arguably a better fit for ISL than SL: in SL adding the missing *M* to the overall precondition $p_0$ is only valid for straight-line code, and not across control flow branches. Intuitively, there is no guarantee that a safe precondition for one path is safe for the other. This is especially the case in the presence of non-determinism or over-approximation of Boolean conditions, where one cannot find definitive predicates to force the analysis down one path. It is thus necessary to *re-execute* the whole procedure on the inferred preconditions, eliminating those that are not safe for all paths. By contrast, in our setting SE-Choice is *sound*, and this re-execution is not needed!

We allow the analysis to abduce information only for *successful* execution; *erroneous* executions have to be *manifest* and realizable using only the information at hand. We do this by requiring *M* to be $\mathsf {emp}$ in SymbExec when applied to error triples. We go even further and require that the implication be in both directions, i.e., that the current state *force* the error – note that if $q\vdash x \mathrel {\not \mapsto }{} * \mathsf {true}$ then there exists *F* such that $x \mathrel {\not \mapsto }{} * F \vdash q$, and similarly for $q\vdash x={\texttt {null}}* \mathsf {true}$. This is a practical choice and only one of many ways to decide *where* to report, trying to avoid blaming the code for issues it did not itself cause. For instance, thanks to this restriction, we do not report on $[x]\!:=10$ (which has error specifications through StoreEr and StoreNull) unless a previous instruction actively invalidated *x*. This choice also chimes well with the fact that the analysis can *start anywhere* in a program and give results relevant to the code analyzed.

Solving the bi-abduction entailment in SymbExec can be done using the techniques developed for SL [11, §3]. We do not detail them here as they are straightforwardly adapted to our simpler setting without inductive predicates.

**Finding a Bug in** client**, Automatically.** We now describe how Pulse automatically finds a proof of the bug in the unnanotated code of client from Fig. 3, by automatically applying the only possible symbolic execution rule at each step. Starting from $\mathsf {emp}$ and going past the first instruction $x\!:=[v]$ requires solving $v \mapsto {u} * F \vdash \mathsf {emp}* M$. The bi-abduction entailment solver then answers with $F=\mathsf {emp}$ and $M=v \mapsto {u}$, yielding the inferred presumption $v \mapsto {u}$ and the next current state $v \mapsto {u} * x = u$. The next instruction is the call to ${\texttt {push\_back(}}v{\texttt {)}}$. For ease of presentation, let us consider this library call as an axiomatized instruction that has been given the specification in Fig. 3. This corresponds to writing a model for it in the analyzer, which is actually the case in the implementation, although the analysis would work equally well if we were to inline the code inside client. Applying SymbExec requires solving the entailment $v \mapsto {a} * a \mapsto {w} * F \vdash v \mapsto {u} * x = u * M$. The solver then answers with the solution $F = (x = u * a = u)$ and $M = u \mapsto {w}$. Finally, the following instance of SE-StoreEr is used to report an error, where $\mathbb {C}= {\texttt {skip}}; x\!:=[v]; {\texttt {push\_back(}}v{\texttt {)}}$ and $q_{ rx } = v \mapsto {a'} * a' \mapsto {w} * a \mathrel {\not \mapsto }{} * x = u * a = u$:**Preliminary Results.** Our analysis handles the examples in this paper, modulo function inlining. While our analysis shows how to derive a sound static analysis from first principles, it does not yet fully exploit the theory, as it does not handle function calls, and in particular *summarization*. Under-approximate triples pave the way towards succinct summaries. However, this is a subtle problem, requiring significant theoretical and empirical work out of the scope of this initial paper.

Pragmatically, we can make Pulse scale by skipping over procedure calls instead of inlining them, in effect assuming that the call has no effect beyond assigning fresh (non-deterministic) values to the return address and the parameters passed by reference – note that such fresh values are treated optimistically by Pulse as we do not know them to be invalid. In theory, this may cause false positives and false negatives, but in practice we observed that such an analysis reports very few issues. For instance, it reports no issues on OpenSSL 1.0.2d (with 8681 C functions) at the time of writing, and only 17 issues on our proprietary C++ codebase of hundreds of thousands of procedures. As expected, the analysis is very fast and scales well (6 s for OpenSSL, running on a Linux machine with 24 cores). Moreover, 30 disjuncts suffice to detect all 17 issues (in comparison, using 20 disjuncts misses 1 issue, while using 100 disjuncts detects no more issues than using 30 disjuncts), and varying loop unrollings between 1–10 has no effect.

We also ran Pulse in production at Facebook and reported issues to developers as they submit code changes, where bugs are more likely than in mature codebases. Over the course of 4 months, Pulse reported 20 issues to developers, of which 15 were fixed. This deployment relies crucially on the begin-anywhere capability: though the codebase in question has 10s of MLOC, analysing a code change starts from the changed files and usually visits only a small fraction of the codebase.

**Under-Approximation in Pulse.** Pulse achieves under-approximate reasoning in several ways. First, Pulse uses the under-approximate Choice, Loop1 and Loop2 rules in Fig. 5 which prune paths by considering one execution branch (Choice) or finite loop unrollings (Loop1 and Loop2). Second, Pulse does not use Alloc2, and thus prunes further paths. Third, Pulse uses under-approximate models of certain library procedures; e.g., the vector::push_back() model assumes the internal array is always deallocated. Finally, our bi-abduction implementation assumes that memory locations are distinct unless known otherwise, thus leading to further path pruning. These choices are all sound thanks to the under-approximate theory of ISL; it is nevertheless possible to make different pragmatic choices.

Although our implementation does not do it, we can use ISL to derive strongest posts for primitive statements, using a combination of their axioms and the Frame, Disj and Exist rules. Given the logic fragment we use (which excludes inductive predicates) and a programming language with Boolean conditions restricted to a decidable fragment, there is likely a bounded decidability result obtained by unrolling loops up to a given bound and then checking the strongest post on each path. However, the ability to under-approximate (by forgetting paths/disjuncts) gives us the leeway to tune a deployment for optimizing the bugs/minute rate: in one experiment, we found that running Pulse on a codebase with 100s kLOC and a limit of 20 disjuncts was $\sim$3.1x user-time faster than running it with a limit of 50 disjuncts, and yet found 97% of the issues found in the 50-disjuncts case.

### Remark 3

Note that although the underlying heaps in ISL grow monotonically, the impact on the size of the manipulated states in our analysis is comparable to that of the original bi-abductive analysis for SL [11]. This is in part thanks to the compositionality afforded by ISL and its footprint property (Theorem 2), especially when individual procedures analyzed are not too big. In particular, the original bi-abduction work for SL already tracks the allocated memory; in ISL we additionally track deallocated memory which is of the same order of magnitude.

## Context, Related Work and Conclusions

Although the foundations of program verification have been mostly developed with correctness in mind, industrial uses of symbolic reasoning often derive value from their deployment as *bug catchers* rather than *provers* of bug absence. There is a fundamental tension in correctness-based techniques, most thoroughly explored in the model checking field, between compact representations versus strength and utility of counter-examples. Abstraction techniques are typically used to increase compactness. This has the undesired side-effect that counter-examples become “abstract”: they may be infeasible, in that they may not actually witness a concrete execution that violates a given property. Using proofs of bugs, this paper aims to provide a symbolic mechanism to express the *definite* existence of a concrete counter-example, without committing to a particular one, while simultaneously enabling sound, compositional, local reasoning. Our working hypothesis is that bugs are a fundamental enough phenomenon to warrant a fundamental compositional theory for reasoning positively about their existence, rather than only being about failed proofs. We hope that future work will explore the practical ramifications of these foundational ideas more thoroughly.

Amongst static bug-catching techniques, there is a dichotomy between the highly scalable, compositional static tools such as Coverity [5], Facebook Infer [18] and those deployed at Google [42], which suffer from false positives as well as negatives, and the under-approximating global bug hunters such as fuzzers [23] and symbolic executors [9], which suffer from scalability limitations but not false positives (at least, ideally). In a recent survey, Godefroid remarks “How to engineer exhaustive symbolic testing (that is, a form of verification) in a cost-effective manner is still an open problem for large applications” [23]. The ability to apply compositional analyses incrementally to large codebases has led to considerable impact that is complementary to that of the global analyses. But, compositional techniques can have less precision compared to global ones: examining all call sites of a procedure can naturally lead to more precise results.

Our illustrative analysis, Pulse, starts from the scalable end of the spectrum and moves towards the under-approximate end. An equally valid research direction would be to start from existing under-approximate analyses and make them more scalable and with lower start-up-cost. There has indeed been valuable research in this direction. For example, SMART [22] tries to make symbolic execution more scalable by using summaries as in inter-procedural static analysis, and UC-KLEE [39] allows symbolic execution to begin anywhere, and thus does not need a complete program. UC-KLEE uses a “lazy initialization” mechanism to synthesize assumptions about data structures; this is not unlike the bi-abductive approach here and in [10]. An interesting research question is whether this similarity can be made rigorous. There are many papers on marrying under- and over-approximation e.g., [1], but they often lack the scalability that is crucial to the impact of modular bug catchers. In general, there is a large unexplored territory, relevant to Godefroid’s open problem stated above, between the existing modular but not-quite-under-approximate bug catchers such as Infer and Coverity, and the existing global and under-approximate tools such as KLEE [8], CBMC [12] and DART [24]. This paper provides not a solution, but a step in the exploration.

Gillian [20] is a platform for developing symbolic analysis tools using a symbolic execution engine based on separation logic. Gillian has C and JavaScript instantiations for precise reasoning about a finite unwinding of a program, similar to symbolic bounded model checking. Gillian’s execution engine is currently exact for primitive commands (it is both over- and under-approximate); however, it uses over-approximate bi-abduction for function calls, and is thus open to false positives (Petar Maksimović, personal communication). We believe Gillian can be modified to embrace under-approximation more strongly, serving as a general engine for proving ISL specifications. Aiming for under-approximate results rather than exact ones gives additional flexibility to the analysis designer, just as aiming for over-approximate rather than exact results does for correctness tools.

Many assertion languages for heap reasoning have been developed, including ones not based on SL (e.g., [3, 27, 31, 46]). We do not claim that, compared to these alternatives, the ISL assertion language in this paper is particularly advantageous for reasoning along individual paths, or exhaustive (but bounded) reasoning about complete programs. Rather, the key point is that our analysis solves abduction and anti-abduction problems, which in turn facilitates its application to large codebases. In particular, as our analysis synthesizes contextual heap assumptions (using anti-abduction), it can begin anywhere in a codebase instead of starting from main(). For example, it can start on a modified function that is part of a larger program: this capability enables continuous deployment in codebases with millions of LOC [18, 34]. To our knowledge, the cited assertion languages have only ever been applied in a whole-program fashion on small codebases (with low thousands of LOC). We speculate that this is not because of the assertion languages *per se*: if methods to solve analogues of abduction and anti-abduction queries were developed, perhaps they too could be applied to large codebases.

It is natural to consider how the ideas of ISL extend to concurrency. The RacerD analyzer [25] provided a static analysis for data races in concurrent programs; this analysis was provably under-approximate under certain assumptions. RacerD was intuitively inspired by concurrent separation logic (CSL [6]), but did not match the over-approximate CSL theory (just as Infer did not match SL). We speculate that RacerD and other concurrency analyses might be seen as constructing proofs in a yet-to-be-defined incorrectness version of CSL, a logic which would aim at finding bugs in concurrent programs via modular reasoning.

Our approach supports reasoning that is local not only in code, but also in state (spatial locality). Spatially local symbolic heap update has led to advances in scalability of global shape analyses of mutable data structures, where heap predicates are modified in-place in a way reminiscent of operational in-place update, and where transfer functions need not track global heap information [44]. Mutable data structures have been suggested as one area where classic symbolic execution has scaling challenges, and SL has been employed with human-directed proof on heap-intensive components to aid the overall scalability of symbolic execution [37]. An interesting question is whether spatial locality in the analysis can benefit scalability of fully automatic, global, under-approximate analyses.

We probed the semantic fundamentals underpinning local reasoning in Sect. 4, including a footprint theorem (Theorem 2) that is independent of the logic. The semantic principles are more deeply fundamental than the surface syntax of the logic. Indeed, in the early days of work on SL, it was remarked that local reasoning flows from locality properties of the semantics, and that separation logic is but one convenient syntax to exploit these [45]. Since then, a number of correctness logics with non-SL syntax have been proposed for local reasoning (e.g., [33] and its references) that exploit the semantic locality of heap update, and it stands to reason that the same will be possible for incorrectness logics.

Relating this paper to the timeline of SL for correctness, we have developed the basic logic (like [36] but under-approximate) and a simple local intra-procedural analysis (like [19] but under-approximate). We have not yet made the next steps to relatively-scalable global analyses [44] or extremely-scalable inter-procedural, compositional ones [11]. These future directions are challenging for theory and especially practice, and are the subject of ongoing and future work.

**Conclusions.** Long ago, Dijkstra (in)famously remarked that “testing can be quite effective for showing the presence of bugs, but is hopelessly inadequate for showing their absence” [17], and he advocated the use of logic for the latter. As noted by others, many of the benefits of logic hold for both bug catching and verification, particularly the ability to cover many states and paths succinctly, even if not the alluring all. But there remains a frustrating division between testing and verification, where e.g., distinct tools are used for each. With more research on the fundamentals of symbolic bug catching and correctness, division may be replaced by unified foundations and toolsets in the future. For under-approximate reasoning in particular, we hope that bug catching eventually becomes more modular, scalable, easier to deploy and with elegant foundations similar to those of verification. This paper presents but one modest step towards that goal.

## References

[1] Albarghouthi A, Gurfinkel A, Chechik M, Flanagan C, König B (2012). From under-approximations to over-approximations and back. Tools and Algorithms for the Construction and Analysis of Systems.

[2] Banerjee A, Naumann DA, Rosenberg S (2013). Local reasoning for global invariants, part I: region logic. J. ACM.

[3] Bansal K, Reynolds A, King T, Barrett C, Wies T, Kroening D, Păsăreanu CS (2015). Deciding local theory extensions via E-matching. Computer Aided Verification.

[4] Berdine J, Calcagno C, O’Hearn PW, de Boer FS, Bonsangue MM, Graf S, de Roever W-P (2006). Smallfoot: modular automatic assertion checking with separation logic. Formal Methods for Components and Objects.

[5] Bessey A (2010). A few billion lines of code later: using static analysis to find bugs in the real world. Commun. ACM.

[6] Brookes S, O’Hearn PW (2016). Concurrent separation logic. SIGLOG News.

[7] Burch, J.R., Clarke, E.M., McMillan, K.L., Dill, D.L., Hwang, L.J.: Symbolic model checking: 10$$^{\wedge }$$20 states and beyond. In: Proceedings of the Fifth Annual Symposium on Logic in Computer Science (LICS 1990), Philadelphia, Pennsylvania, USA, 4–7 June 1990, pp. 428–439 (1990). 10.1109/LICS.1990.113767

[8] Cadar, C., Dunbar, D., Engler, D.R.: KLEE: unassisted and automatic generation of high-coverage tests for complex systems programs. In: 8th USENIX Symposium on Operating Systems Design and Implementation, OSDI 2008, San Diego, California, USA, 8–10 December 2008, Proceedings, pp. 209–224 (2008). http://www.usenix.org/events/osdi08/tech/full_papers/cadar/cadar.pdf

[9] Cadar C, Sen K (2013). Symbolic execution for software testing: three decades later. Commun. ACM.

[10] Calcagno C, Distefano D, O’Hearn PW, Yang H, Nielson HR, Filé G (2007). Footprint analysis: a shape analysis that discovers preconditions. Static Analysis.

[11] Calcagno C, Distefano D, O’Hearn PW, Yang H (2011). Compositional shape analysis by means of bi-abduction. J. ACM.

[12] Clarke E, Kroening D, Lerda F, Jensen K, Podelski A (2004). A tool for checking ANSI-C programs. Tools and Algorithms for the Construction and Analysis of Systems.

[13] Cohen E, Moskal M, Schulte W, Tobies S, Touili T, Cook B, Jackson P (2010). Local verification of global invariants in concurrent programs. Computer Aided Verification.

[14] Costanzo D, Shao Z, Jhala R, Igarashi A (2012). A case for behavior-preserving actions in separation logic. Programming Languages and Systems.

[15] Cousot, P., Cousot, R.: Abstract interpretation: a unified lattice model for static analysis of programs by construction or approximation of fixpoints. In: POPL, pp. 238–252 (1977). 10.1145/512950.512973

[16] Cousot P, Cousot R, Horspool RN (2002). Modular static program analysis. Compiler Construction.

[17] Dijkstra EW (1976). A Discipline of Programming.

[18] Distefano D, Fähndrich M, Logozzo F, O’Hearn PW (2019). Scaling static analyses at Facebook. Commun. ACM.

[19] Distefano D, O’Hearn PW, Yang H, Hermanns H, Palsberg J (2006). A local shape analysis based on separation logic. Tools and Algorithms for the Construction and Analysis of Systems.

[20] Fragoso Santos, J., Maksimović, P., Ayoun, S., Gardner, P.: Gillian, part i: a multi-language platform for symbolic execution. In: Proceedings of the 41st ACM SIGPLAN International Conference on Programming Language Design and Implementation (PLDI 2020), London, UK, 15–20 June 2020 (2020). 10.1145/3385412.3386014

[21] Gardner PA, Maffeis S, Smith GD (2012). Towards a program logic for javascript. SIGPLAN Not..

[22] Godefroid, P.: Compositional dynamic test generation. In: Proceedings of the 34th ACM SIGPLAN-SIGACT Symposium on Principles of Programming Languages, POPL 2007, Nice, France, 17–19 January 2007, pp. 47–54 (2007). 10.1145/1190216.1190226

[23] Godefroid P (2020). Fuzzing: hack, art, and science. Commun. ACM.

[24] Godefroid, P., Klarlund, N., Sen, K.: DART: directed automated random testing. In: Proceedings of the ACM SIGPLAN 2005 Conference on Programming Language Design and Implementation, Chicago, IL, USA, 12–15 June 2005, pp. 213–223 (2005). 10.1145/1065010.1065036

[25] Gorogiannis N, O’Hearn PW, Sergey I (2019). A true positives theorem for a static race detector. PACMPL.

[26] Hoare CAR (1969). An axiomatic basis for computer programming. Commun. ACM.

[27] Holík L, Hruška M, Lengál O, Rogalewicz A, Vojnar T, Bouajjani A, Monniaux D (2017). Counterexample validation and interpolation-based refinement for forest automata. Verification, Model Checking, and Abstract Interpretation.

[28] Ishtiaq, S.S., O’Hearn, P.W.: BI as an assertion language for mutable data structures. In: Proceedings of the 28th ACM SIGPLAN-SIGACT Symposium on Principles of Programming Languages, POPL, pp. 14–26. Association for Computing Machinery, New York (2001). 10.1145/360204.375719

[29] Kassios IT (2011). The dynamic frames theory. Formal Asp. Comput..

[30] Leino KRM, Clarke EM, Voronkov A (2010). Dafny: an automatic program verifier for functional correctness. Logic for Programming, Artificial Intelligence, and Reasoning.

[31] Mathur U, Murali A, Krogmeier P, Madhusudan P, Viswanathan M (2020). Deciding memory safety for single-pass heap-manipulating programs. Proc. ACM Program. Lang..

[32] McPeak, S., Gros, C., Ramanathan, M.K.: Scalable and incremental software bug detection. In: Joint Meeting of the European Software Engineering Conference and the ACM SIGSOFT Symposium on the Foundations of Software Engineering, ESEC/FSE 2013, Saint Petersburg, Russian Federation, 18–26 August 2013, pp. 554–564 (2013). 10.1145/2491411.2501854

[33] Murali A, Peña L, Löding C, Madhusudan P (2020). A first-order logic with frames. Programming Languages and Systems.

[34] O’Hearn, P.W.: Continuous reasoning: scaling the impact of formal methods. In: Proceedings of the 33rd Annual ACM/IEEE Symposium on Logic in Computer Science, LICS 2018, Oxford, UK, 09–12 July 2018, pp. 13–25 (2018). 10.1145/3209108.3209109

[35] O’Hearn PW (2019). Incorrectness logic. Proc. ACM Program. Lang..

[36] O’Hearn P, Reynolds J, Yang H, Fribourg L (2001). Local reasoning about programs that alter data structures. Computer Science Logic.

[37] Pirelli, S., Zaostrovnykh, A., Candea, G.: A formally verified NAT stack. In: Proceedings of the 2018 Afternoon Workshop on Kernel Bypassing Networks, KBNets@SIGCOMM 2018, Budapest, Hungary, 20 August 2018, pp. 8–14 (2018). 10.1145/3229538.3229540

[38] Raad, A., Berdine, J., Dang, H.H., Dreyer, D., O’Hearn, P., Villard, J.: Technical appendix (2020). http://plv.mpi-sws.org/ISL/

[39] Ramos, D.A., Engler, D.R.: Under-constrained symbolic execution: correctness checking for real code. In: 2016 USENIX Annual Technical Conference, USENIX ATC 2016, Denver, CO, USA, 22–24 June 2016 (2016). https://www.usenix.org/conference/atc16/technical-sessions/presentation/ramos

[40] Raza, M., Gardner, P.: Footprints in local reasoning. Logical Methods Comput. Sci. **5**(2) (2009). 10.2168/LMCS-5(2:4)2009

[41] Reynolds, J.C.: Separation logic: a logic for shared mutable data structures. In: Proceedings of the 17th Annual IEEE Symposium on Logic in Computer Science. LICS 2002, pp. 55–74. IEEE Computer Society, Washington, DC (2002). http://dl.acm.org/citation.cfm?id=645683.664578

[42] Sadowski C, Aftandilian E, Eagle A, Miller-Cushon L, Jaspan C (2018). Lessons from building static analysis tools at Google. Commun. ACM.

[43] de Vries E, Koutavas V, Barthe G, Pardo A, Schneider G (2011). Reverse hoare logic. Software Engineering and Formal Methods.

[44] Yang H, Gupta A, Malik S (2008). Scalable shape analysis for systems code. Computer Aided Verification.

[45] Yang H, O’Hearn P, Nielsen M, Engberg U (2002). A semantic basis for local reasoning. Foundations of Software Science and Computation Structures.

[46] Yorsh G, Rabinovich AM, Sagiv M, Meyer A, Bouajjani A (2007). A logic of reachable patterns in linked data-structures. J. Log. Algebr. Program..

